# Quantitative trait loci associated with flowering time in wild chrysanthemum (*Chrysanthemum seticuspe*)

**DOI:** 10.1270/jsbbs.25074

**Published:** 2026-07-01

**Authors:** Katsuhiko Sumitomo, Sachiko Isobe, Miyuki Kunihisa, Hideki Hirakawa, Kenta Shirasawa, Masafumi Yagi, Tamotsu Hisamatsu, Yoshihiro Nakano, Fumiya Taniguchi, Kazuki Matsubara, Yohei Higuchi

**Affiliations:** 1 Institute of Vegetable and Floriculture Science, NARO, Fujimoto, Ibaraki 305-8519, Japan; 2 Kazusa DNA Research Institute, 2-6-7 Kazusa-Kamatari, Kisarazu, Chiba 292-0818, Japan; 3 Institute of Fruit Tree and Tea Science, NARO, Fujimoto, Ibaraki 305-8605, Japan; 4 Institute of Crop Science, NARO, 2-1-2 Kannondai, Ibaraki 305-8518, Japan; 5 Graduate School of Agricultural and Life Sciences, The University of Tokyo, 1-1-1 Yayoi, Bunkyo, Tokyo 113-8657, Japan

**Keywords:** critical daylength, photoperiodism, QTL mapping

## Abstract

The mechanisms of flowering-time (FT) regulation in cultivated chrysanthemum (*Chrysanthemum morifolium*) are essential for breeders owing to their significant effects on cultivation. The wild diploid *Chrysanthemum seticuspe* has the same short-day flowering habits as cultivated chrysanthemum. We investigated an F_2_ segregating population derived from crosses of accessions with differences in FT for quantitative trait locus (QTL) mapping. Three major QTLs for FT were mapped, each overlapping with QTLs for other flowering traits: one QTL associated with a longer critical daylength and two QTLs linked to the ability to flower rapidly following floral transition under short-day conditions, one of which also contributes to flowering competence at high temperatures. These traits collectively influence the regulation of FT. Altered expression patterns of circadian-clock-related genes in SoS4-4, the early-flowering parental line, suggested altered rhythmicity of the circadian clock. Circadian-clock-related genes and a photoreceptor gene were also found in the FT QTL interval. These genes may be responsible for altering the circadian clock and causing variation in FT by changing the critical daylength.

## Introduction

Chrysanthemum species are herbaceous perennials distributed in East Asia. Most are short-day (SD) plants, blooming in autumn under SD conditions. The transition from vegetative to reproductive phase is crucial in the plant life cycle. Timely autumn flowering increases reproductive success before winter and is vital for adaptation in temperate climates. Cultivated chrysanthemum (*Chrysanthemum morifolium* Ramat.), a hexaploid species, is economically important as an ornamental crop, with flowering controlled to meet market demands. Flowering can be controlled by manipulating daylength using blackout curtains and light exposure in protected cultivation. In Japan, a wide range of cultivars that flower from May to December are grown in open fields (Kawata and Funakoshi 1988). Understanding the mechanism controlling flowering time (FT) is key to chrysanthemum production and breeding.

Flowering in chrysanthemum is complex and is influenced by multiple environmental factors. Daylength is a major factor (Kawata and Funakoshi 1988, Langton 1977, Okada 1957). Flowering is regulated by a daylength threshold, the ‘critical daylength’, and under natural conditions is promoted as daylength shortens. Cultivars with a longer critical daylength flower earlier. Other factors such as ambient temperature, temperature memory (the temperature of prior growing conditions), nutrition and plant hormones affect chrysanthemum flowering (Harada and Nitsch 1959, Hisamatsu *et al.* 2017, Schwabe 1950, Tjia *et al.* 1969). It is proposed that FT is determined mainly by the combination of daylength and temperature, because it was explained by the relationship between the photoperiodic and temperature responses among cultivars with a wide range of FTs (Kawata and Funakoshi 1988).

In many plant species, genetic factors regulating FT are identified by quantitative trait locus (QTL) analysis (Koornneef *et al.* 2004, Matsubara and Yano 2018). Genetic mapping studies of cultivated chrysanthemum have detected loci controlling FT (Su *et al.* 2024, van Geest *et al.* 2017), but analysis is challenging owing to the hexaploid genome. To avoid this problem, the wild diploid *Chrysanthemum seticuspe* (Maxim.) Hand.-Mazz. (2*n* = 18) is used in genomic and molecular studies as a model species (Hirakawa *et al.* 2019, Nakano *et al.* 2019, 2021). It has similar growth and SD-flowering habits to cultivated chrysanthemum, flowering from October to November (National BioResource Project [Chrysanthemum]: https://shigen.nig.ac.jp/chrysanthemum/). Using *C. seticuspe* for QTL studies allows efficient genetic analysis of FT and helps us understand how environmental factors influence flowering. Here we explored potential mechanisms underlying chrysanthemum FT regulation, such as variations in response to daylength and candidate genes.

## Materials and Methods

### Plant materials

We grew F_2_ plants developed from a cross between the *C. seticuspe* lines SoS4-4 and XMRS10. SoS4-4 was developed from the early-flowering accession Matsukawa1 (Fukai and Miyatake 2005) via four generations of selfing. XMRS10 is a late-flowering line developed from the accession AEV02 via five generations of selfing (Nakano *et al.* 2019). XMRS10 was provided by National BioResource Project Chrysanthemum (https://shigen.nig.ac.jp/chrysanthemum). One F_1_ plant was self-pollinated to generate the F_2_ population. A total of 186 F_2_ individuals, each parental line and F_1_ were grown as stock plants in plastic pots (12-cm i.d., 1 seedling per pot) containing a commercial horticultural soil (Kureha-Engei-Baido; Kureha Chemical Co. Ltd., Tochigi, Japan). The plants were maintained in the vegetative state in a glasshouse (heated at <18°C and ventilated at >25°C) under natural photoperiod plus a 6-h night break (22:00–04:00) to prevent floral transition. The night-break treatment was conducted using white fluorescent lamps (EFR25ED/22; Toshiba Lighting & Technology Corporation, Kanagawa, Japan) at a photon flux density (PFD) of 3.3 μmol m^–2^ s^–1^. Stock plants were exposed to natural low temperatures (<10°C) to break dormancy in an unheated plastic greenhouse during winter (January–March). Rooted cuttings were prepared from these stock plants for phenotypic studies of flowering traits at the NARO Institute of Vegetable and Floriculture Sciences (NIFTS; Ibaraki, Japan; 36°2ʹ46ʺN, 140°5ʹ55ʺE).

### Phenotypic data collection

#### Flowering time

FT was determined in open fields under natural temperatures and daylength. On 4 July 2017, five rooted cuttings per genotype were planted at 15-cm intervals in two rows 45 cm apart. Ten days after planting, the plants were pinched at the internode between the third and fourth leaves from the base to encourage the growth of lateral shoots. This technique increased growth uniformity by synchronizing the outgrowth of shoots. We recorded the date of first anthesis (i.e., when the ray florets of the terminal flower became vertically oriented) of each plant. FT was scored as the number of days after 4 July. In 2018, one rooted cutting per genotype was planted. Details are shown in Table 1.

#### Reaction time

Reaction time (RT) is the number of days from SD perception to flowering. We investigated RT under optimal temperature and SD conditions for flowering promotion (RT_opt_). On 3 August 2017, four rooted cuttings per genotype were planted at 16 cm × 16 cm in a plastic house. Plants were pinched 10 days after planting. Lateral shoots, except for the axillary shoot of the topmost leaf on the main stem, were removed 2 weeks later. Plants were grown with a daily 6-h night break to prevent flowering until 19 September 2017. Under the following natural SD conditions (photoperiod 12.25–10.00 h) to 26 November, we recorded the date of first anthesis of each plant. The greenhouse was heated at <20°C and ventilated at >23°C. RT_opt_ per genotype was calculated as the difference between the end date of night-break treatment (19 September) and anthesis date (Fig. 1). In 2018 and 2020, two rooted cuttings per genotype were planted. In 2020, plants were grown under ≤11-h photoperiod by closing the blackout curtains from 1700 to 0600 after the end date of night-break treatment.

### Reaction time under high temperature

High temperature (HT) delays chrysanthemum flowering (Nakano *et al.* 2013, Whealy *et al.* 1987). We investigated its effect on RT in the glasshouse in summer, where restricted ventilation raised temperatures. On 20 June 2017, three rooted cuttings per genotype were planted at 12 cm × 12 cm. Plants were pinched 10 days after planting. Lateral shoots, except for the axillary shoot of the topmost leaf on the main stem, were removed 2 weeks later. Plants were grown with a daily 6-h night break until 6 July 2017. Plants were then grown under the following HT conditions in summer with 11-h SD conditions achieved by closing blackout curtains from 1700 to 0600. The windows were closed at <22°C to maintain HT. We recorded the date of first anthesis of each plant. RT under HT (RT_HT_) per plant was the number of days from RT_opt_ to the delayed anthesis date (Fig. 1). In 2018, the windows were closed at <25°C. The average temperature during the experimental period in the glasshouse was 26.2°C in 2017 and 28.1°C in 2018 (Table 1).

### Reaction time under 13-h photoperiod

We investigated the effect of suboptimal daylength on RT. On 30 July 2018, two rooted cuttings per genotype were planted in plastic pots (30-cm i.d., 8 cuttings per pot) containing a commercial horticultural soil in the glasshouse. Plants were pinched 10 days after planting. Lateral shoots, except for the axillary shoot of the topmost leaf on the main stem, were removed 2 weeks later. Plants were grown vegetatively with a daily 6-h night break until 14 September 2018. Under the following suboptimal 13-h photoperiod from 14 September to 5 December, we recorded the date of first anthesis of each plant. To create a 13-h photoperiod, we used incandescent lamps to supply predawn lighting. The glasshouse was maintained at optimal temperature (20–23°C). RT under 13-h photoperiod (RT_13h_) per genotype was the number of days from RT_opt_ to the delayed anthesis date (Fig. 1). In spring 2020, plants were planted at 14 cm × 14 cm in planting beds (90 cm W, 600 cm L, 18 cm D) in the glasshouse, and a 13-h photoperiod was created by closing blackout curtains from 1700 to 0600 (Table 1).

Spearman’s correlation coefficients for each trait in 2017 and 2018 were analysed using MS-Excel. The normality of data distribution for each trait was assessed by the Shapiro–Wilk test (*P* < 0.05) in EZR v. 1.54 software (Kanda 2013).

### Linkage map construction and QTL analysis

Genomic DNA was extracted from shoot tips (30 mg fresh weight) with a DNeasy Plant Mini Kit (Qiagen, Hilden, Germany) according to the manufacturer’s instructions. ddRAD-Seq libraries were constructed with restriction enzymes *Pst*I and *Msp*I and sequenced by MiSeq at a read length of 251 nt. The reads were mapped onto the Gojo-0_v1 sequence (Nakano *et al.* 2021) in Bowtie2 software (Langmead and Salzberg 2012), and variant calls were performed in SAMtools v. 0.1.19 (Li *et al.* 2009a) and VarScan 2.3 (Koboldt *et al.* 2012) software as described by Shirasawa *et al.* (2016). Candidate SNPs were filtered under the following conditions: exclude indels, minimum depth = 5, minimum quality = 10, max missing = 0.5, minor allele frequency = 0.2, polymorphic between parental lines.

SNP markers with ≤10% missing genotypes were selected and divided into nine groups by alignment to chromosomes on Gojo-0_v1 for map construction. A linkage map was constructed in Lep-MAP3 software (Rastas 2017). The Filtering2 function of Lep-MAP3 was not used, because there were many SNPs with distorted distributions on chromosomes 1, 2, 5, 7 and 8. The SeparateChromosomes2 function was used to cluster groups at a logarithm of odds (LOD) score of 30. The OrderMarkers2 function was used to order the mapped markers within each linkage group (LG) by maximizing the best-likelihood positions of the markers and to compute genetic distance, with 20 cM as the cut-off threshold for LGs. LG numbering and direction were defined with reference to the reported chromosomes (Nakano *et al.* 2021). Relationships between SNP markers and traits were analysed by the non-parametric Kruskal–Wallis (K-W) test in MapQTL 6.0 software (van Ooijen 2009). Loci with larger K* values than the significance level (*P* < 0.00001, set as 0.05/6824 markers) were regarded as putative QTLs.

### Genomic analysis

We searched non-synonymous sequence variants within flowering-related genes for QTL intervals. Whole-genome shotgun sequencing of the parental lines was performed on a 100-bp paired-end Illumina HiSeq 4000 sequencer. Sequencing reads were mapped onto Gojo-0_v1 in Bowtie2. Variant calling was based on the mapping result in SAMtools 0.1.19 software and variants were filtered in VarScan v. 2.3 software. Effects of variants on gene function were predicted in SnpEff v. 4.0 software (Cingolani *et al.* 2012). Of the genes harbouring SNPs with high or moderate effects of non-synonymous sequence variants on genes and proteins in Gojo-0_v1 genomic regions corresponding to QTL intervals, we identified flowering-related genes as *C. seticuspe* genes described in Hirakawa *et al.* (2019) and *C. seticuspe* homologues of Arabidopsis photoperiod and circadian clock genes as described in the FLOR-ID database of FT gene networks (Bouché *et al.* 2016).

### Gene expression analysis

We quantified 24-h expression profiles of circadian-clock-related genes—*CsLHY* (*LATE ELONGATED HYPOCOTYL*, CsG_LG1.g30264.1, GenBank Accession No. AB733625), *CsTOC1* (*TIMING OF CAB EXPRESSION 1*, CsG_LG6.g49179.1, AB733626) and *CsGI* (*GIGANTEA*, CsG_LG8.g71869.i1, AB733627)—in XMRS10 and SoS4-4 under continuous dark conditions (DD). Plants were grown in plastic pots (6-cm i.d., 1 rooted cutting per pot) and maintained in a growth chamber under a 16-h photoperiod under white fluorescent lamps at 70 μmol m^–2^ s^–1^ PFD (23°C, 80% relative humidity) for 6 weeks. At the end of the 6 weeks, plants were transferred to DD (23°C, 80% relative humidity). One fully expanded leaf per plant was harvested hourly over 24 h and immediately frozen in liquid nitrogen. Total RNA was extracted with an RNeasy Plant Mini Kit (Qiagen K.K., Tokyo, Japan) and treated with RNase-free DNase (Qiagen K.K.). From each sample, 100 ng of total RNA was reverse-transcribed using a PrimeScript RT Master Mix Perfect Real Time Kit (Takara Bio Inc., Shiga, Japan), with three biological replicates. The resulting cDNA was diluted 1:10 with distilled water. Quantitative PCR was performed using 1 μL cDNA template in 10 μL with TB Green Premix Ex Taq II Tli RNase H Plus (Takara Bio Inc.) on a Thermal Cycler Dice Real Time System III (Takara Bio Inc.) for 1 min of denaturation at 95°C, followed by 40 cycles of 95°C for 5 s and 60°C for 30 s, with the primers listed in Supplemental Table 1. The raw data were normalized to the level of expression of *CsACTIN* (CsG_LG2.g19422.i1, AB770470). The cycle threshold (CT) of *CsACTIN* did not differ among the samples. ΔCT of the genes of interest was calculated as CT – CT_CsACTIN_. The maximum ΔCT value was used as a reference and set at 1.0. Then ΔΔCT of each gene was calculated as ΔCT_sample_ – ΔCT_reference_. The data are presented as 2^–ΔΔCT^ (Livak and Schmittgen 2001).

### Data availability statement

The data that support the findings of this study are available from the corresponding author, upon reasonable request.

## Results

### Flowering time and flowering traits

The range of phenotypic variation within the F_2_ population was more or less continuous between the parental lines (Fig. 2). Distributions were strongly correlated between years, except RT_13h_ at 0.52 (Fig. 2D). For every flowering trait, SoS4-4 had smaller values than XMRS10, and F_1_ plants had smaller values than the midpoint between the parents, except RT_HT_ in 2017 (Fig. 2C). RT_opt_, RT_HT_ and RT_13h_ showed transgressive segregation. FTs were later in 2018 than in 2017. Distributions of RT_opt_ were similar between 2017 and 2018 but were slightly smaller in 2020 (Fig. 2B). SoS4-4 flowered early under both HT and a 13-h photoperiod (Fig. 2C, 2D). The distribution varied between years: RT_HT_ was larger in 2018 because the greenhouse temperature was higher (Table 1). However, data were correlated between years.

Spearman’s correlation analysis suggested that 7 of the 9 possible trait pairs had significant (*P* < 0.001) correlations (Table 2). FT showed a positive correlation with the other three flowering traits. Two trait pairs, FT and RT_opt_ in 2017 and 2018 (ρ = 0.71 and 0.51, respectively) and FT and RT_13h_ in 2018 (ρ = 0.59) displayed moderate correlation coefficients, while a trait pair, FT and RT_HT_ displayed a weak correlation coefficient in 2017 and 2018 (ρ = 0.31 and 0.36, respectively).

### Linkage map construction and QTL for flowering traits

After trimming and removal of low-quality sequences, we obtained ~2.2 million high-quality reads per F_2_ plant. The reads were mapped onto the Gojo-0_v1 sequence with average mapping alignment rates of 91.2%. Between SoS4-4 and XMRS10, a total of 10,100 SNPs were identified. Among these, we used the genotypes of the F_2_ population at 6,824 polymorphic SNP sites to construct a genetic map. The map consisted of 780 loci and covered 878.50 cM in 9 LGs, consistent with the haploid number of the species, with an average locus interval of 1.13 cM (Table 3). Segregation distortion was significantly higher in LGs 1, 2, 7 and 8, where SoS4-4 homozygotes were reduced, and in LG 5, where XMRS10 homozygotes were reduced (Supplemental Table 2), indicating a biological bias in meiosis or chromosome pairing.

The K-W test mapped 11 QTLs for four flowering traits on LGs 2, 4, 6 and 8 (Fig. 3) but none on LG 1, 3, 5, 7 or 9 (Supplemental Fig. 1). It detected four significant QTLs for FT on LGs 2, 4 and 6. Detection of *qFT2.1*, *qFT4.1* and *qFT6.2* in both years indicates that they determined FT consistently between years. *qFT6.2* was a major QTL with a very high K* value. At all four QTLs, the average FT of F_2_ plants representative of the SoS4-4 allele was earlier than that of plants carrying the XMRS10 allele (Table 4). *qFT6.2* had the largest effect: FT became earlier with the number of SoS4-4 alleles. FT was 3.9 days earlier in 2017 and 3.4 days earlier in 2018 in SoS4-4 *qFT6.2* homozygotes than in heterozygotes. On the other hand, there was no difference between SoS4-4 *qFT2.1* and *qFT4.1* homozygotes and heterozygotes.

Two QTLs controlling RT_opt_—*qRT2.1* and *qRT4.1*—were consistently detected in 3 years; for each one, SoS4-4 contributed the allele for smaller RT_opt_. *qRT8.1* was detected in only 2017, and the XMRS10 allele contributed to smaller RT_opt_. A major QTL for RT_HT_ was detected on LG 4 in 2017. The significant interval was broad and had two K* peaks, so we divided it into QTLs *qHT4.1* and *qHT4.2* at 45.4 cM. Two corresponding QTLs were detected in 2018. The allele for shorter RT_HT_ originated from SoS4-4. *q13H6.2*, a major QTL with a remarkably high K* value, for RT_13h_ was detected on LG 6 in both years. *q13H6.1*, a minor QTL, was detected at the left end of LG 6 in 2020. The allele for shorter RT_13h_ was contributed by SoS4-4: RT_13h_ became shorter as the number of SoS4-4 alleles of *q13H6.2* increased. Three FT QTLs overlapped with QTLs for other flowering traits: *qFT2.1* with *qRT2.1*; *qFT4.1* with *qRT4.1* and *qHT4.1*; and *qFT6.2* with *q13H6.2*. At each QTL colocalization, the allele effects on phenotypes were similar: phenotypic values became smaller as the number of SoS4-4 alleles in *qFT6.2* and *q13H6.2* increased.

### Candidate genes

*qFT2.1*, *qFT4.1* and *qFT6.2* were detected consistently in both years, indicating that each region is likely to contain a gene related to FT. We determined that a ~193-Mbp physical region (46,017,926–239,207,382 bp) on chromosome 2 of Gojo-0_v1, corresponding to the overlapping interval (32.0–33.6 cM) for *qFT2.1* in 2017 and 2018, potentially contained the candidate gene for *qFT2.1*. Of the 4,399 genes in the region, 1,208 harbour non-synonymous base variants between the parental lines (Supplemental Table 3). Among them, *CsCYC2a* (Hirakawa *et al.* 2019), *CsFTL1* (Oda *et al.* 2012) and a putative circadian-clock-related gene, *CsRVE2a* (Hirakawa *et al.* 2019), were related to flowering in previous reports and in FLOR-ID (Table 5). Similarly, nine flowering-related genes were discovered in the region (273,664,494–312,897,337 bp) of *qFT4.1*, being homologous to Arabidopsis *EMF2*, *FVE*, *GAI* and *HDA5*. The region (2,676,270–326,802,638 bp) corresponding to the *qFT6.2* genomic interval covered almost all of chromosome 6. Nevertheless, 25 candidate genes, including putative circadian-clock-related genes (*CsLNK1*, *CsPRR7*, *CsTOC1* and *CsELF3*) and a photoreceptor gene (*CsPHYA*), were discovered.

### Gene expression analysis

Mutations in the circadian clock alter FT in rice and Arabidopsis (Hori *et al.* 2016, Johansson and Staiger 2015). We investigated the expression of circadian-clock-related genes *CsLHY*, *CsTOC1* and *CsGI*, putative orthologues of Arabidopsis core clock component genes *LHY/CCA1*, *TOC1/PRR1* and *GI*, respectively (Hirakawa *et al.* 2019, Oda *et al.* 2017, 2020). *CsLHY* and *CsGI* are located on chromosomes 1 and 8, respectively. *CsTOC1* is located in the interval of *qFT6.2* and has non-synonymous base variants between parents (Table 5). Although the transcript abundance of these genes appeared to show no substantial differences between XMRS10 and SoS4-4 (Supplemental Fig. 2), direct comparisons of gene expression levels were avoided (Fig. 4) in view of the stability of housekeeping gene expression and variations in biological backgrounds. Under continuous dark conditions (DD), *CsLHY* expression started increasing at subjective dawn (i.e., when the plant subjectively perceives the start of the light period due to being entrained under the 16-h light/8-h dark cycle before transferring to DD) and peaked between 9 and 10 h in XMRS10, but between 11 and 12 h in SoS4-4, indicating a phase shift of the expression pattern (Fig. 4A). *CsTOC1* and *CsGI* in SoS4-4 also showed a slightly later phase shift (Fig. 4B, 4C), but the sampling period was too short. Although *CsTOC1* expression was increasing in XMRS10 at 24 h, it might increase in SoS4-4 after 24 h. *CsGI* expression peaked at 24 h in XMRS10 but was still increasing in SoS4-4.

## Discussion

The main objective of this study was to elucidate the genetic architecture of FT regulation in *C. seticuspe*. Three major QTLs for FT were detected consistently in 2 years. Nevertheless, minor QTLs might remain unmapped owing to the small sample size, the use of a relatively conservative threshold, and higher segregation distortion on several chromosomes. The three major QTLs show that mechanisms of FT regulation are genetically dissected into three major flowering components. We also investigated correlation coefficients and QTL colocalization between FT and other flowering traits, and the results explained the physiological role of each FT QTL.

FT QTL on chromosome 6, *qFT6.2*, colocalized with *q13H6.2*, the QTL for flowering competence under a 13-h photoperiod. Flowering is sensitive to daylength, and critical daylength varies among *C. seticuspe* accessions (Oda *et al.* 2012). The early-flowering SoS4-4 has a longer critical daylength than the late-flowering XMRS10. This trait contributes to the flowering competence under a 13-h photoperiod. Thus, the QTL colocalization on chromosome 6 indicates that the longer critical daylength contributed to early FT. Floral transition and early flower bud development are thought to occur in August, when daylength is shortening but still suboptimal for most *C. seticuspe* accessions (13:00–13:59 h at NIFTS, Tsukuba). The early-flowering SoS4-4 perceives this daylength as a short day, and the signal triggers the transition from vegetative to reproductive growth. In addition, the smaller RT_opt_ of SoS4-4 indicates a faster flower bud development after floral transition, which contributes to an early FT. *qFT2.1* and *qFT4.1* are associated with the smaller RT_opt_ of SoS4-4 and colocalize with QTLs for RT_opt_, *qRT2.1* and *qRT4.1*, respectively. RT_opt_, the number of days to flowering under optimal temperature and photoperiod, indicates the endogenous responsiveness of flowering to inductive conditions, namely the competence to flower quickly following floral transition. Moreover, the overlap of *qFT4.1* with both *qHT4.1* and *qRT4.1* indicates that *qFT4.1* acts as a flowering promoter with HT tolerance in SoS4-4. HT conditions delay flowering in *C. seticuspe*, mainly via the inhibition of capitulum development from inflorescence meristem formation to floret growth (Nakano *et al.* 2013). Flowering of late-flowering XMRS10 was delayed under HT and thus was putatively thermosensitive, whereas SoS4-4 was less sensitive. QTL analysis revealed that early FT in SoS4-4 is regulated by three flowering components: the component of flowering competence under suboptimal daylength and two components of the promotion of flower bud development, one of which is tolerant to HT. Results also suggest that *C. seticuspe* knows when to trigger flowering by monitoring daylength as a flowering cue in summer when it is still suboptimal. Because daylength is an extremely stable signal, chrysanthemums can use it as their main cue for stable reproductive growth. However, the FT phenotypic value varied slightly between 2017 and 2018 (Fig. 2A). HT conditions suppress flowering even after reproductive growth has been triggered, so the average FT was delayed in 2018, when average temperatures were higher (Table 1).

Photoperiodic flowering is regulated by the balance between florigen and anti-florigen in *C. seticuspe* (Higuchi *et al.* 2013, Oda *et al.* 2012). The anti-florigen gene *CsAFT* is induced under long-day (LD), whereas *CsAFT* is downregulated and the florigen gene *CsFTL3* is upregulated under SD. Genes located within the FT QTL may play a role in regulating the expression of *CsAFT* and *CsFTL3*. The overlap of *qFT2.1* with *qRT2.1* indicates that *qFT2.1* is associated with flowering competence under SD conditions. *CsFTL1*, a candidate gene in *qFT2.1*, is a paralogue of the main florigenic gene *CsFTL3* (Oda *et al.* 2012). However, *CsFTL1* is an unlikely responsible gene for *qFT2.1*, because it contributes to floral induction under LD (Higuchi *et al.* 2013). HT suppresses flowering in *C. seticuspe* through downregulation of *CsFTL3* (Nakano *et al.* 2013). The candidate genes in *qFT4.1* may be involved in thermoregulated suppression of *CsFTL3*, because *qFT4.1* overlapped with *qHT4.1*, which is associated with flowering competence under HT. The eight genes, except for CsG_LG4.g62650.1, are putatively orthologous to genes involved in suppression of *FT* in Arabidopsis (Li *et al.* 2008). It remains to be clarified whether these genes are involved in the suppression of *CsFTL3* by HT in chrysanthemum.

*qFT6.2* was associated with setting critical daylength. *CsAFT* expression is upregulated when photoreception is synchronized with the endogenous photosensitive phase (Higuchi *et al.* 2013). The setting of the photosensitive phase is controlled by the circadian clock; e.g., the critical daylength was shortened with the delay of the photosensitive phase in *CsGI*-overexpressing plants (Oda *et al.* 2020), and conversely, a longer critical daylength was correlated with an early-generated photosensitive phase and a shortened circadian rhythmicity, as suggested by leaf movements, in chrysanthemum (Takahashi *et al.* 2025). The longer critical daylength in SoS4-4 may be mediated by an early photosensitive phase. The expression pattern of the *CsLHY* in XMRS10 is similar to that previously reported for the wildtype ZBL1 (Oda *et al.* 2017) and can be considered typical. In contrast, the expression pattern in SoS4-4 may represent a variant type, as indicated by the slight delay of the peak phase of circadian-clock-related genes. Circadian-clock-related genes (*CsLNK1*, *CsPRR7*, *CsTOC1* and *CsELF3*) and a photoreceptor gene (*CsPHYA*) in *qFT6.2* may be responsible for altering the circadian clock in SoS4-4. These results suggest that variations in the circadian clock are involved in the diversity of critical daylength in *C. seticuspe*.

Two RT_opt_ QTLs, *qRT2.1* and *qRT4.1*, collocate with previously reported QTLs for the number of days to flowering under SD conditions in cultivated chrysanthemum (van Geest *et al.* 2017). Wild diploid *C. seticuspe* and cultivated chrysanthemum may share flowering-related genes on chromosomes 2 and 4. However, QTLs associated with a longer critical daylength were detected on different chromosomes: on chromosome 6 (*q13H6.2*) in *C. seticuspe* but on chromosome 2 in cultivated chrysanthemum (Takahashi *et al.* 2025). This indicates that the genes responsible for critical daylength may not be shared between *C. seticuspe* and cultivated chrysanthemum, and that breeders can develop novel early-flowering cultivars with longer critical daylength in cultivated chrysanthemum by using *q13H6.2* through interspecific hybridization, genetic modification and genome editing.

In conclusion, QTL analysis revealed the genetic architecture that regulates FT of *C. seticuspe*. FT was predominantly determined by the effect of variations in critical daylength, which was controlled by a single QTL. In addition, endogenous SD responsiveness, controlled by two QTLs, was also involved in determining FT. The possibility that an altered circadian clock results in the longer critical daylength and early FT in *C. seticuspe* is consistent with a report on cultivated chrysanthemum (Takahashi *et al.* 2025), but the gene responsible for the longer critical daylength may be different, as the QTLs are located on different chromosomes between *C. seticuspe* and cultivated chrysanthemum. The use of the diploid *C. seticuspe* proved effective for studying the mechanism of photoperiodic flowering in the highly heterozygous, outcrossing hexaploid chrysanthemum. This study provides insights into the function of candidate genes and engineering the traits of cultivated chrysanthemums.

## Author Contribution Statement

KaS, Conceptualization, Methodology, Validation, Investigation, Resources, Data curation, Visualization, Project administration, Funding acquisition, Writing—original draft, review & editing. SI, HH, KeS, Methodology, Funding acquisition, Supervision, Investigation, Data curation, Writing—original draft, review & editing. MK, Methodology, Supervision, Writing—review & editing. MY, TH, YN, FT, KM, YH, Supervision, Writing—review & editing.

## Supplementary Material

Supplemental Figures

Supplemental Tables

## Figures and Tables

**Fig. 1.**
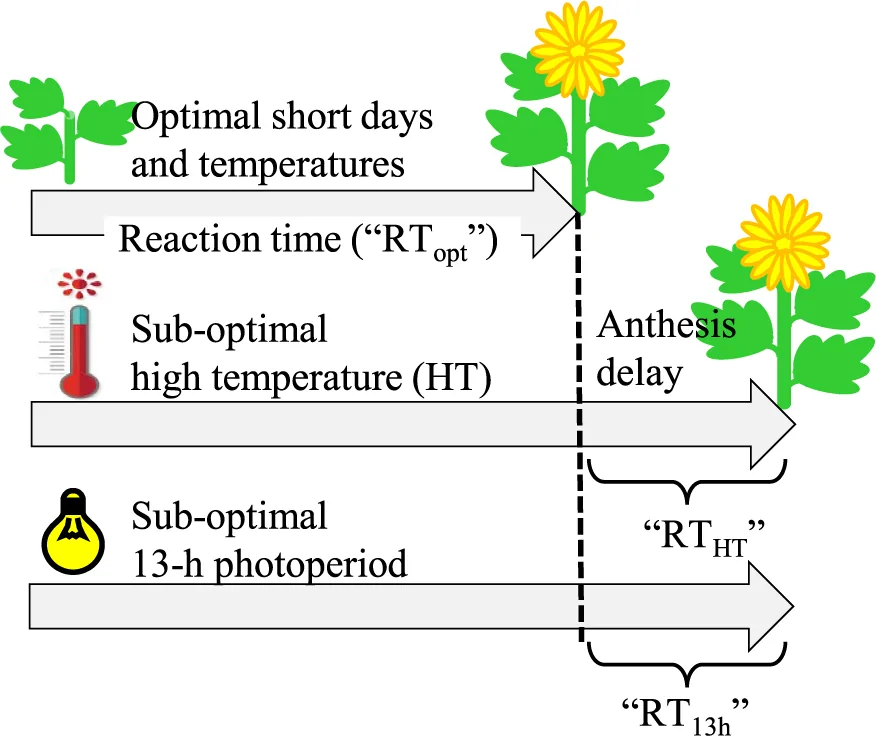
Measurement of flowering traits of the SoS4-4 × XMRS10 F_2_ population. Reaction time (RT_opt_), number of days to flowering under optimal temperature and short-day conditions; RT_HT_, flowering delay caused by high temperature; RT_13h_, flowering delay by suboptimal 13-h photoperiod.

**Fig. 2.**
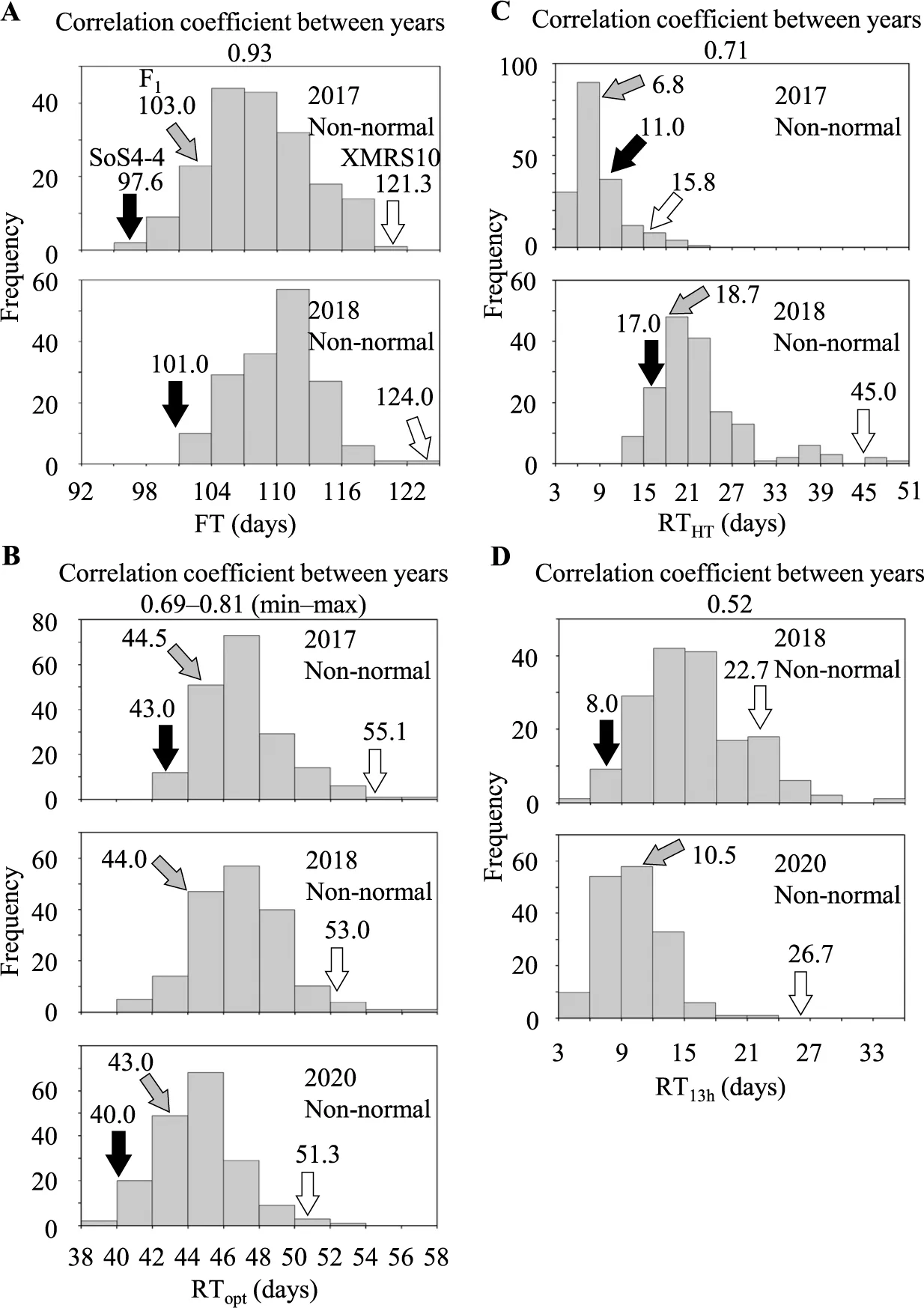
Distribution of phenotypes in the SoS4-4 × XMRS10 F_2_ population. (A) FT: number of days from planting to flowering under natural conditions in the open field; no data were available for F_1_ in 2018. (B) RT_opt_: number of days to flowering under optimal temperature and short-day conditions; no data were available for SoS4-4 in 2018. (C) RT_HT_: increase in number of days to flowering caused by high temperature. (D) RT_13h_: increase in number of days to flowering under 13-h photoperiod; no data were available for F_1_ in 2018 or for SoS4-4 in 2020. Distribution type by Shapiro–Wilk test is shown in each panel. Correlation coefficient of distributions between years is shown at the top of each panel. 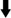 SoS4-4; 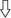 XMRS10; 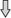 F_1_.

**Fig. 3.**
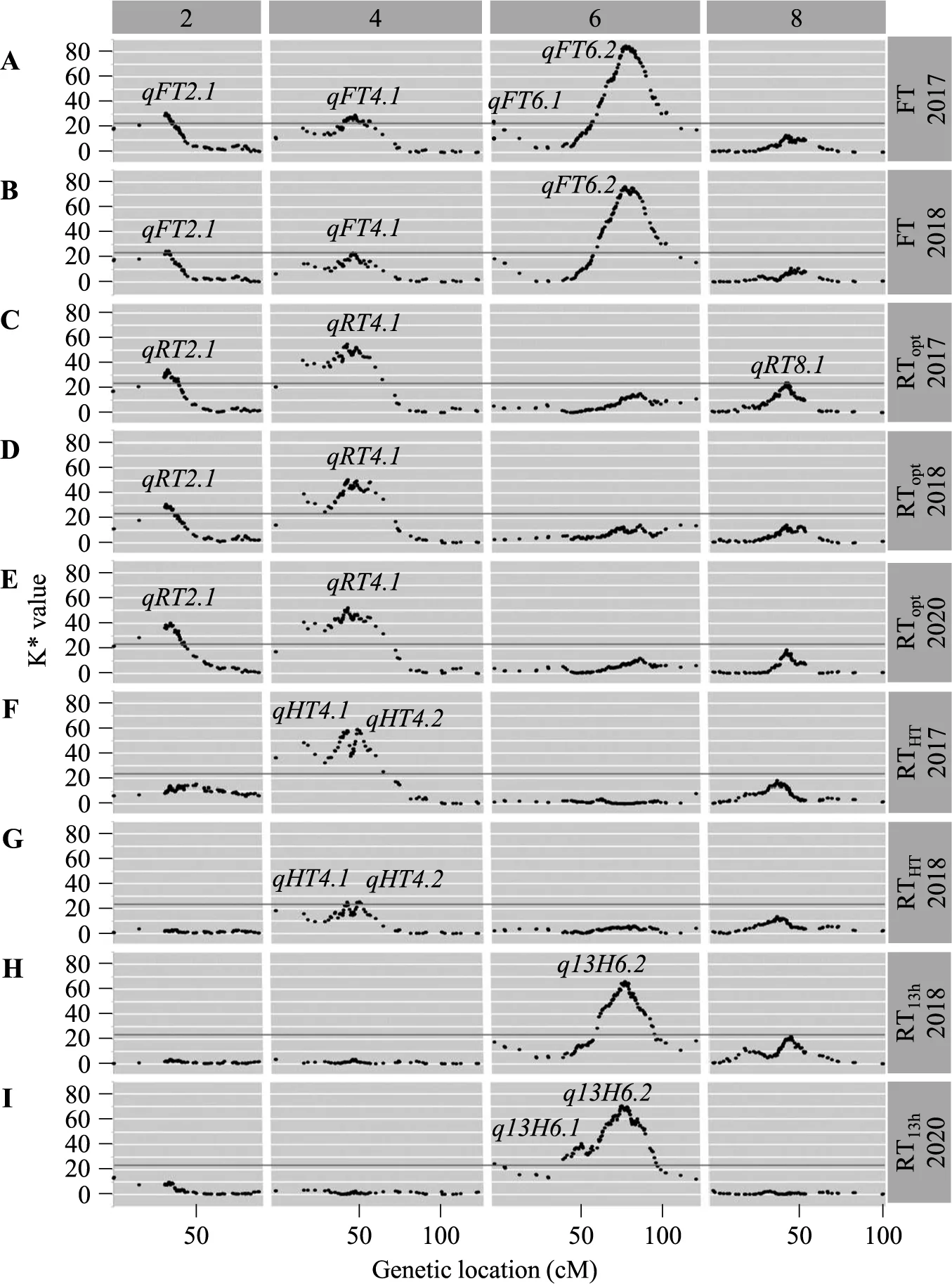
QTL K* values for flowering time and flowering-related traits shown along linkage groups 2, 4, 6 and 8 (marked at the top). (A, B) FT in 2017 and 2018. (C–E) RT_opt_ in 2017, 2018 and 2020. (F, G) RT_HT_ in 2017 and 2018. (H, I) RT_13h_ in 2018 and 2020. QTLs (*P* < 0.00001) were detected by Kruskal–Wallis test at the threshold shown by horizontal grey lines (K* = 23.1).

**Fig. 4.**
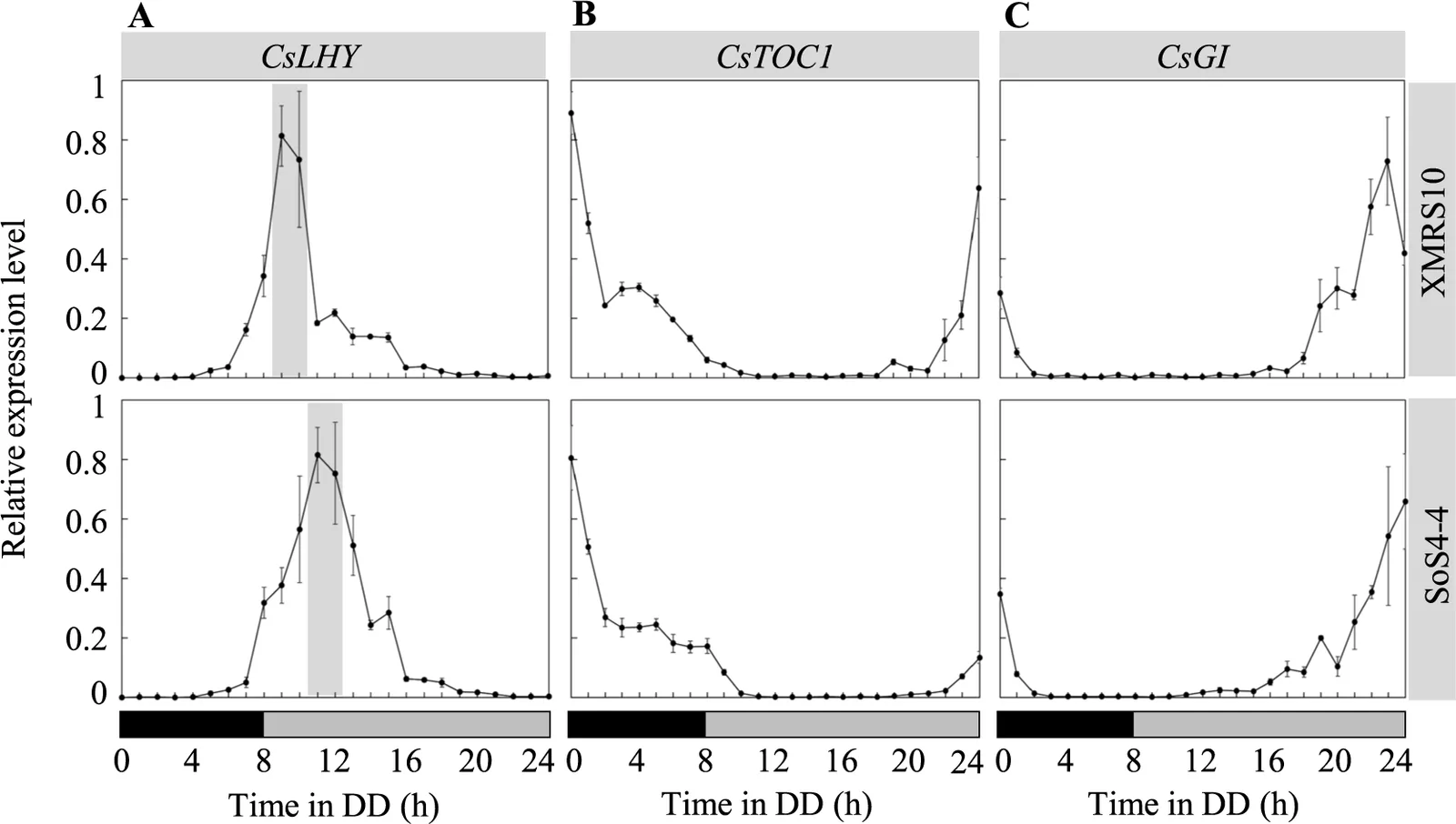
Expression of circadian-clock-related genes (A) *CsLHY*, (B) *CsTOC1* and (C) *CsGI* in early-flowering SoS4-4 and late-flowering XMRS10 parental *C. seticuspe* lines under continuous dark (DD) condition. Plants were grown under 16-h light/8-h dark before the experiment. Leaves were harvested hourly for gene expression analyses. 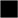 Time of subjective dark; 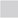 Time of subjective light. Expression levels were normalized to *CsACTIN*. Values are means ± SE (*n* = 3).

**Table 1.** Design and planting information for phenotyping

Trait	Year	Date of transplanting (MM/DD)	Date of starting treatment (MM/DD)	Daylength (h, Min–Max)	Temperature (Min–Max)	Growth condition	*n*
FT	2017	07/04	–	10.40–14.58	Avg 21.8°C (2.3–35.0°C)	Field	5
FT	2018	07/04	–	10.40–14.58	Avg 23.2°C (3.9–37.4°C)	Field	1
RT_opt_	2017	08/03	09/19	10.00–12.25	20–23°C	Glasshouse	4
RT_opt_	2018	08/03	09/19	10.00–12.25	20–23°C	Glasshouse	2
RT_opt_	2020	08/11	09/23	10.00–11.00*^a^*	20–23°C	Glasshouse	2
RT_HT_	2017	06/20	07/06	11.00*^a^*	Avg 26.2°C	Glasshouse	3
RT_HT_	2018	06/20	07/06	11.00*^a^*	Avg 28.1°C	Glasshouse	2
RT_13h_	2018	07/30	09/14	13.00*^a^*	20–23°C	Glasshouse	2
RT_13h_	2020	03/10	04/10	13.00*^a^*	20–23°C	Glasshouse	2

*^a^* Daylength was controlled using blackout curtains or supplementary lighting.

**Table 2.** Spearman’s rank correlation coefficients between phenotypes in the SoS4-4 × XMRS10 F_2_ population

Year	Traits*^a^*	FT	RT_opt_	RT_HT_
2017	RT_opt_	0.71*		
	RT_HT_	0.31*	0.30*	
2018	RT_opt_	0.51*		
	RT_HT_	0.36*	0.01	
	RT_13h_	0.59*	0.14	0.27*

*^a^* FT, flowering time; RT_opt,_ days to flower under optimal SD and temperature conditions; RT_HT_, flowering delay caused by high temperature; RT_13h_, flowering delay caused by 13-h photoperiod. * Significant at *P* < 0.001.

**Table 3.** Summary of the genetic linkage map constructed with the F_2_ population from the SoS4-4 × XMRS10 cross

Linkage group	Number of loci		Length (cM)	Average bin interval (cM)
	SNPs	Bin		
1*	437	58	79.36	1.37
2*	933	71	88.83	1.25
3	695	87	89.22	1.03
4	658	69	123.12	1.78
5*	971	109	82.06	0.75
6	901	117	123.00	1.05
7*	643	84	88.98	1.06
8*	854	89	103.83	1.17
9	732	96	100.11	1.04
All	6824	780	878.50	1.13

* Segregation distortion was higher in LGs 1, 2, 5, 7 and 8.

**Table 4.** Genomic intervals indicated in the Kruskal–Wallis test as associated with the genetic determination of quantitative traits of flowering time and flowering-related traits in the SoS4-4 × XMRS10 F_2_ population

Trait*^a^*	QTL	Linkage group	Year	Interval (cM)	Mean value*^b^*		
					SoS4-4	Heterozygote	XMRS10
FT	*qFT2.1*	2	2017	31.2–36.1	107.1	107.8	111.9
			2018	32.0–33.6	110.4	110.3	113.6
	*qFT4.1*	4	2017	41.6–57.3	107.7	108.7	112.7
			2018	46.5–46.8	110.7	110.8	114.1
	*qFT6.1*	6	2017	0.0	108.2	110.1	112.1
	*qFT6.2*	6	2017	59.6–104.8	104.1	108.0	113.3
			2018	62.7–104.8	107.3	110.7	114.6
RT_opt_	*qRT2.1*	2	2017	31.2–40.1	45.7	46.6	48.4
			2018	31.2–36.1	46.1	46.3	48.6
			2020	15.6–42.8	44.0	44.1	46.2
	*qRT4.1*	4	2017	16.6–65.2	46.1	46.8	49.7
			2018	16.7–65.2	46.0	46.8	49.8
			2020	16.7–65.2	43.7	44.6	47.2
	*qRT8.1*	8	2017	44.9–46.5	50.4	46.6	47.8
RT_HT_	*qHT4.1*	4	2017	0.0–45.4	7.6	7.9	12.4
			2018	43.2–43.5	22.1	20.6	29.4
	*qHT4.2*	4	2017	45.4–65.2	7.5	7.9	12.3
			2018	49.2–51.6	21.8	20.7	29.6
RT_13h_	*q13H6.1*	6	2018	0.0	9.6	10.2	12.2
	*q13H6.2*	6	2018	62.7–97.6	11.6	14.5	19.5
			2020	42.0–98.7	7.3	9.4	12.7

*^a^* FT, flowering time; RT_opt_, days to flower under optimal SD and temperature conditions; RT_HT_, flowering delay caused by high temperature; RT_13h_, flowering delay caused by 13-h photoperiod. *^b^* Mean phenotypic value of allele of SNP marker detected as best linked with the QTL.

**Table 5.** Flowering-related genes in genomic intervals of major flowering-time QTLs

QTL	Gene on Gojo-0_v1	Arabidopsis homologue	Previously reported chrysanthemum gene
*qFT2.1*	CsG_LG2.g12748.1	*FT*	*CsFTL1*, *CmFTL1* (Oda *et al.* 2012)
	CsG_LG2.g32477.1	*RVE2*	*CsRVE2a* (Hirakawa *et al.* 2019)
	CsG_LG2.g38814.1	*CYC2*	*CsCYC2a* (Hirakawa *et al.* 2019) *CmCYC2a* (Huang *et al.* 2016) *ClCYC2a* (Zhang *et al.* 2024)
*qFT4.1*	CsG_LG4.g58216.1	*HDA5*	
	CsG_LG4.g58311.1	*HDA5*	
	CsG_LG4.g58315.1	*HDA5*	
	CsG_LG4.g58373.1	*HDA5*	
	CsG_LG4.g58376.1	*HDA5*	
	CsG_LG4.g58392.1	*HDA5*	
	CsG_LG4.g59314.1	*EMF2*, *CYR1*	
	CsG_LG4.g62062.1	*FVE*, *MSI4*	
	CsG_LG4.g62650.1	*GAI*, *RGA2*	
*qFT6.2*	CsG_LG6.g05334.1	*SPA3*	*CsSPA3* (Hirakawa *et al.* 2019)
	CsG_LG6.g05549.1	*SPL3*	
	CsG_LG6.g06567.1	*GA2ox1*	*CmGA2ox* (Lyu *et al.* 2022)
	CsG_LG6.g11837.1	*LNK1/2*	*CsLNK1* (Hirakawa *et al.* 2019)
	CsG_LG6.g12204.1	*MSI1*	
	CsG_LG6.g12223.1	*MSI1*	
	CsG_LG6.g14873.1	*PHYA*	*CsPHYA* (Hirakawa *et al.* 2019)
	CsG_LG6.g19116.1	*TOE1*, *RAP2.7*	
	CsG_LG6.g19334.1	*PRR7*	*CsPRR7* (Hirakawa *et al.* 2019)
	CsG_LG6.g20751.1	*GA20ox1*	*CmGA20ox* (Suh *et al.* 2023) *CmGA20ox2* (Yu *et al.* 2022)
	CsG_LG6.g27433.1	*TPS1*	
	CsG_LG6.g29932.1	*RGL2*	
	CsG_LG6.g29933.1	*RGL2*	
	CsG_LG6.g29934.1	*RGL2*	
	CsG_LG6.g29938.1	*RGL2*	
	CsG_LG6.g30342.1	*FCA*	
	CsG_LG6.g38092.1	*ELF3*	*CsELF3* (Hirakawa *et al.* 2019) *ClELF3* (Fu *et al.* 2014)
	CsG_LG6.g41100.1	*JMJ32*, *JMJD5*	
	CsG_LG6.g49123.1	*CDF3*	
	CsG_LG6.g49179.1	*TOC1/PRR1*	*CsTOC1* (Hirakawa *et al.* 2019) *CmTOC1* (Higuchi *et al.* 2012)
	CsG_LG6.g50194.1	*FUL*, *AGL8*	*CsAFL1* (Oda *et al.* 2012) *CmAFL1* (Li *et al.* 2009b)
	CsG_LG6.g58587.1	*EMF2*, *CYR1*	
	CsG_LG6.g59991.1	*BAF60*, *CHC1*	
	CsG_LG6.g62380.1	*TPL*	
	CsG_LG6.g63706.1	*GA3OX1*	*CmGA3ox* (Yang *et al.* 2014) *CmGA3ox1* (Yu *et al.* 2022)
