# Supplementary material for: Quantitative trait loci associated with flowering time in wild chrysanthemum (Chrysanthemum seticuspe)
Source: Breed Sci. 2026 Jul 1;76(3):263–74. doi: 10.1270/jsbbs.25074 (PMC13709645; doi:10.1270/jsbbs.25074)
Supplement: Supplementary file 1 [file 76_263_s1.pdf]

Sumitomo et al./Breeding Science
doi: 10.1270/jsbbs.25074

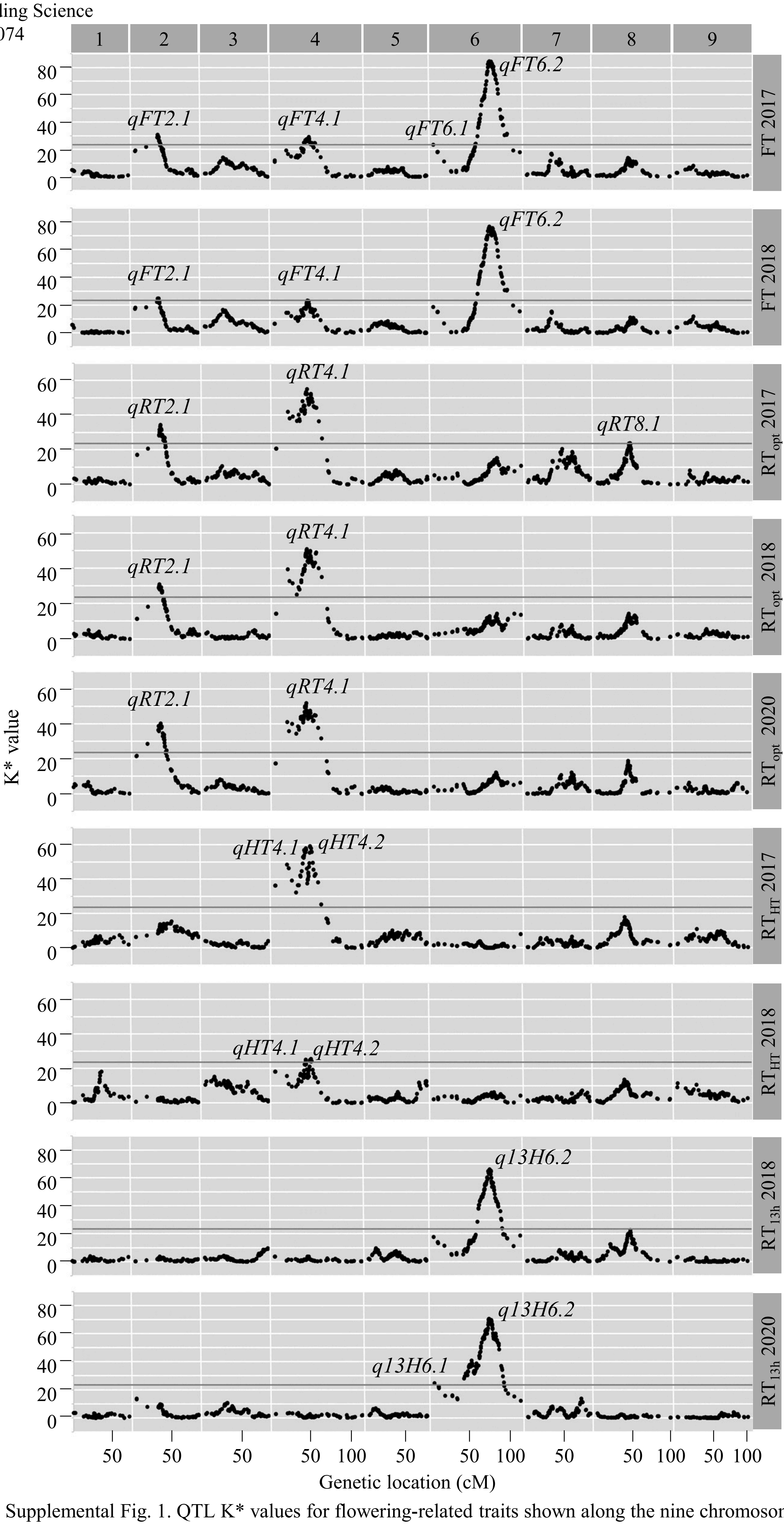

Supplemental Fig. 1. QTL K* values for flowering-related traits shown along the nine chromosomal linkage groups (designated at the top). QTLs ($p < 0.00001$) were detected by Kruskal–Wallis test. The threshold is shown by horizontal black lines (K* value = 23.1).

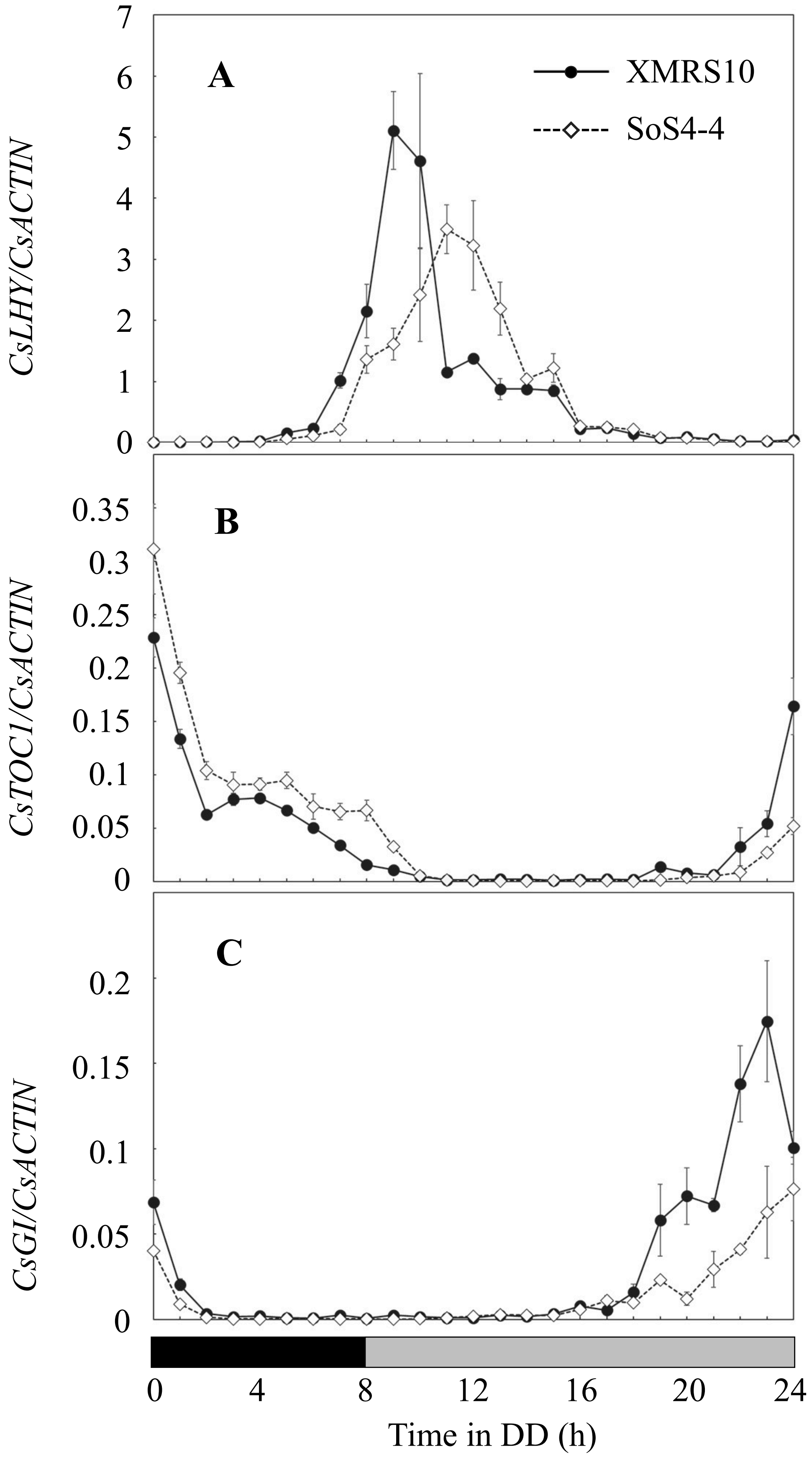

Supplemental Fig. 2. Expression of (A) *CsLHY*, (B) *CsTOC1*, and (C) *CsGI* in early-flowering SoS4-4 and late-flowering XMRS10 parental *C. seticuspe* lines under continuous dark (DD). Plants were grown under 16-h light/8-h dark before the experiment. Leaves were harvested hourly for gene expression analysis. ■ Time of subjective dark; ■ Time of subjective light. Transcript levels were compared directly after normalization against a *CsACTIN* loading standard. Threshold cycle numbers in *CsACTIN* were 21.3–22.9 and 21.5–23.0 in XMRS10 and SoS4-4, respectively. Values are means ± SE (n=3).
